# From Binding Sites to Target Genes: Reframing Human microRNA Target Prediction

**DOI:** 10.3390/ncrna12050040

**Published:** 2026-10-19

**Authors:** Stephanie Sammut, Elissavet Zacharopoulou, Dimosthenis Tzimotoudis, Panagiotis Alexiou

**Affiliations:** Centre for Molecular Medicine and Biobanking, University of Malta, MSD 2080 Msida, Malta; stephanie.sammut@um.edu.mt (S.S.);

**Keywords:** miRNA, machine learning, miRNA target prediction, benchmarking

## Abstract

MicroRNA (miRNA) target prediction is often treated as a single problem, yet it encompasses two distinct computational tasks: identifying whether a miRNA can bind a particular target RNA binding site, and predicting the change in gene expression that follows from miRNA perturbation. Here, we trace the evolution of the field of miRNA target prediction in humans from early seed- and conservation-based algorithms, to deep learning models trained on large-scale interaction datasets. We note that early frameworks obscured binding site recognition and gene-level regulation by aggregating binding site-level scores into gene-level predictions, whereas newer experimental and computational advances have enabled a clearer separation of the tasks. Deep learning has substantially advanced miRNA binding site prediction, but progress in miRNA target gene prediction remains constrained by limited perturbation data, inconsistent data processing and annotations, heterogeneous definitions of regulatory response, uneven predictor coverage, and the absence of comprehensive benchmarks. We also highlight the importance of negative-example construction, which can introduce dataset-specific biases and inflate apparent model performance. We propose two complementary benchmarked community challenges: the prediction of miRNA–binding site interactions, and the genome-wide prediction of miRNA-mediated changes in target gene expression in defined biological contexts. Treating these as distinct computational tasks, while recognising their biological interdependence, would enable more meaningful evaluation of methodological advances and provide a stronger foundation for modelling miRNA-mediated regulatory networks.

## 1. Mapping the microRNA Target Prediction Field

MicroRNAs (miRNAs) are short non-coding RNAs that guide Argonaute-containing silencing complexes to target RNA transcripts, reducing protein output via mRNA destabilisation, translational repression, or both [1,2]. As individual miRNAs can regulate hundreds of transcripts, and a single transcript may be regulated by multiple miRNAs, miRNAs form part of a broad and complex post-transcriptional regulatory layer involved in various fundamental biological processes, including development [3,4,5], and aging [6]. Beyond their roles in normal human physiology, miRNA dysregulation is associated with various diseases including cancer [7], cardiovascular disease [8], neurological disorders [9], and immunoregulatory and inflammatory conditions [10]. This regulatory scope has made miRNAs attractive as biomarkers of disease and as therapeutic targets or agents, via miRNA mimics and inhibitors [11].

Human miRNAs and their targets do not bind with full complementarity. Instead, the AGO protein preferentially presents selected regions of the miRNA for recognition of the target RNA, whereas much of the remaining miRNA is sequestered within the protein and becomes accessible only after conformational changes induced by target binding [12]. This allows miRNAs to recognise binding sites via partial complementarity, with uneven importance across the miRNA–binding site duplex. However, this partial complementarity also makes the rules of miRNA binding difficult to define and model. Pairing to a short ‘seed’ sequence near the 5′ end of the miRNA is an important determinant of many binding interactions, but the presence of a seed match is neither sufficient nor necessary for repression of the target RNA [13,14].

Therefore, identifying a potential binding site is not the same task as predicting a functional miRNA–gene interaction. This distinction motivates the central argument of this perspective. The field of ‘miRNA target prediction’ has historically pursued two related but distinct goals:

(1) To model and predict the potential of a miRNA to bind a candidate target site.
(2) To model and predict the effect of miRNA binding on the expression of a target gene.

We refer to these goals as ‘miRNA binding site prediction’ and ‘miRNA target gene prediction’, respectively. Throughout this article, we use ‘miRNA target prediction’ as an umbrella term for both challenges, while arguing that their differences are central to understanding the historical development and future direction of the field.

## 2. A History of the miRNA Target Prediction Field(s)

The importance of the tasks associated with ‘miRNA target prediction’ was almost immediately recognised following the discovery of mammalian miRNAs [15] and their importance as post-transcriptional regulators [16]. The first generation of ‘miRNA target prediction’ algorithms included miRanda [17], TargetScan [18], PicTar [19], and DIANA-microT [20], among others [21]. These algorithms involved searching transcript sequences, particularly 3′ untranslated regions (3′UTRs), for candidate binding sites and assigning scores based on features such as seed match type, evolutionary conservation, sequence context, and thermodynamic stability. This design enabled interpretable target prediction methods that are useful for generating hypotheses, while also encouraging prediction frameworks based on scoring candidate binding sites and aggregating scores into transcript- or gene-level predictions. This approach effectively conflated the tasks of ‘miRNA binding site prediction’ and ‘miRNA target gene prediction’ by assuming that improved identification of binding sites would translate directly into improved prediction of transcript-level repression.

In 2008, large-scale transcriptomic and proteomic perturbation studies [22,23] generated datasets that subsequently became standard benchmarks for evaluating ‘miRNA target gene prediction’ algorithms. These experiments used early high-throughput methods, namely microarrays and quantitative proteomics, to measure transcriptome- and proteome-wide changes following the overexpression of individual miRNAs in cultured cells. In 2009, a benchmarking effort of the contemporary state of the art on the proteomic perturbation experiment dataset [23] highlighted the difficulty of the ‘miRNA target gene prediction’ task [21]. At a precision level of 50%, i.e., half of the predicted targets being correct and half incorrect, the two best algorithms (TargetScan, DIANA-microT) showed around 10–15% sensitivity, meaning they identified only 10–15% of all true functional targets. This means that the best algorithms of the time could retrieve only approximately one in eight real functional targets, while also predicting an equal number of false positives.

Perhaps unsurprisingly, the widespread adoption of high-throughput sequencing technologies produced a new wave of ‘miRNA target gene prediction’ algorithms [24], which extended earlier approaches by incorporating additional features and expanding the regions considered for binding, including coding sequences (CDSs) alongside 3′UTRs. The introduction of high-throughput crosslinking and immunoprecipitation methods expanded the available evidence by enabling the transcriptome-wide identification of Argonaute-associated RNA binding sites. Techniques such as HITS-CLIP [25] and PAR-CLIP [26] provided large-scale maps of AGO occupancy. Because these methods identify Argonaute-bound RNA regions rather than direct miRNA–target pairs, computational inference was required to assign specific miRNAs to their corresponding binding sites. This substantially expanded the number of candidate miRNA binding sites available for computational modelling.

These developments broadened the scope of prediction and allowed the inclusion of interactions beyond strictly conserved seed matches. The derived datasets improved prediction model performance; for example, DIANA-microT-CDS [27], evaluated on the same proteomic perturbation experiment benchmarking datasets from 2008 [23], reported almost double the sensitivity from that reported in the benchmarking effort in 2009 [21], to approximately 20%, at 50% precision. Despite being far from perfect, with one in five functional targets retrieved for an equal number of false positives, the improvement within a few years was notable. Yet, this second generation of algorithms mainly refined the existing scoring framework rather than redefining the underlying prediction problem. Candidate binding sites were still identified using engineered features, machine learning approaches were used to estimate the contribution of individual features, and transcript-level predictions were still generated through the aggregation of site-level scores.

A major turning point occurred in 2013 with the introduction of the CLASH method, which produced the first direct evidence of interaction between miRNAs and their binding sites via chimeric reads [28]. Similar techniques, namely CLEAR-CLIP [29] and chimeric eCLIP [30], further improved capture efficiency and strengthened the evidence for widespread non-canonical binding, which suggests that miRNA recognition extends beyond canonical seed-matching rules. This experimental development was promising for the ‘miRNA binding site prediction’ task because it provided large-scale examples of true miRNA–binding site interactions. Coupled with advances in deep learning and GPU-accelerated computing, this gave rise to a third generation of prediction algorithms based on Deep Neural Networks, including TargetScan-CNN [31], cnnMirTarget [32], TargetNet [33], miRBind [34], a CNN-based model [35], and an attention-based model [36], amongst others. These algorithms were included in miRBench, a standardised benchmarking framework for miRNA binding site prediction algorithms [37]. By defining a common prediction task and evaluation framework, miRBench helped formalise ‘miRNA binding site prediction’ as a distinct computational problem from ‘miRNA target gene prediction’. An important limitation of chimeric read techniques is that while they provide evidence of observed miRNA–binding site interactions, they do not define reliable non-binding pairs. Consequently, despite greatly increasing the availability of positive binding interaction data, high-throughput experiments have not solved the problem of generating reliable negative examples for supervised miRNA target prediction. Instead, negative examples must be constructed computationally. Several approaches have been adopted, each of which is limited by its own assumptions. Examples include sequence shuffling [38,39], which creates artificial miRNAs or targets; sampling and filtering scarce experimentally tested non-interacting miRNA–mRNA pairs [33,40] or random transcript sampling [32,36], which may each introduce bias relating to sequence composition of different genomic features; or pairing miRNAs to alternative targets from the positive class [34,35,41], which treats unrecovered interactions from the same cellular context as negatives, despite the possibility that they were not detected due to biological or experimental limitations.

The choice of the method for negative example generation matters because artificial negatives may differ from positives in ways that are not biologically meaningful, allowing models to learn dataset-specific patterns rather than true miRNA targeting rules. In 2025, we discovered that a simple miRNA frequency class bias was affecting the most commonly used binding site-level training sets and benchmarks to date, leading to the overestimation of model performance and poor generalisation to independent datasets [37]. The bias originated from the method used to generate negative examples. Improving the negative example generation procedure and correcting the resulting bias allowed us to develop a substantially stronger miRNA target site prediction algorithm with markedly improved generalisation across datasets [37], while also showing promise as a foundation for addressing the ‘miRNA target gene prediction’ problem [42].

For the task of ‘miRNA target gene prediction’, several machine learning approaches have also been proposed with miRAW [40], TargetScan [31], MirTarget v4.0 [43], TEC-miTarget [44], microT-CNN [45], and miRBind2-3UTR [42] providing prominent examples. Existing approaches have explored diverse modelling strategies. While miRBind2-3UTR exploits transfer learning from large target-site datasets, miRAW, TargetScan, MirTarget v4.0, TEC-miTarget and microT-CNN predict target-site activity before aggregating site-level predictions across 3′ UTRs into a gene- or transcript-level score. miRBind2-3UTR learns directly from the 3′ UTR sequence without handcrafted features. microT-CNN extends prediction beyond 3′ UTRs to include coding sequences (CDSs). These methods also differ in the evidence used for training, including experimentally validated interactions (e.g., miRTarBase and DIANA-TarBase), miRNA perturbation RNA-seq datasets, CLIP-based binding assays (e.g., HITS-CLIP, PAR-CLIP, CLASH and CLEAR-CLIP), AGO RNA Bind-n-Seq binding affinity measurements, or combinations of these experimental data. Their prediction objectives also vary: some methods predict the probability of a target gene for a given miRNA (miRAW, MirTarget v4.0, TEC-miTarget, microT-CNN), whereas others claim to directly predict the magnitude of miRNA-mediated gene repression (TargetScan, miRBind2-3UTR).

Despite the growing number of predictors, there is still no comprehensive evaluation of their relative performance; benchmarking of ‘miRNA target gene prediction’ methods remains lacking. Progress has been hindered by the limited availability of perturbation experiments, inconsistencies in their processing, heterogeneous gene and miRNA annotations, inconsistencies in the definition and direction of functional responses, and differences in predictor score scales. In addition, the fair comparison of prediction methods is complicated by substantial differences in the breadth of miRNAs and genes covered by different predictors. Compounding these challenges, functional repression is highly dependent on cellular context. The effect of a miRNA–gene interaction may vary across tissues or cell types because of differences in their expression, target transcript isoform usage, RNA-binding proteins, and the broader regulatory environment. Consequently, perturbation measurements obtained in one cellular system may not generalise directly to another, limiting their suitability for benchmarking ‘miRNA target gene prediction’ methods. As a result, advances in ‘miRNA target gene prediction’ have lagged behind those achieved for ‘miRNA binding site prediction’.

## 3. The Future of the Field

While deep learning has transformed ‘miRNA binding site prediction’, its impact on ‘miRNA target gene prediction’ remains difficult to quantify. While the experimental datasets discussed above capture different aspects of miRNA regulation, including AGO occupancy, direct miRNA–binding site interactions, and changes in transcript or protein abundance following miRNA perturbation, these outcomes are not interchangeable prediction units. While the underlying biological processes are interconnected, distinguishing their computational prediction objectives enables each task to be defined and evaluated independently. Given the rapid growth in the availability of experimental datasets, together with continued advances in deep learning methods, we propose two key challenges for the ‘microRNA target prediction’ field over the coming years:

(1) ‘miRNA binding site prediction’: Given a miRNA sequence and a candidate binding site sequence, predict the probability of binding. This challenge was codified in the miRBench benchmark [37], and may be further strengthened through the careful incorporation of additional experimental datasets. An important future extension would be the inclusion of experimental measurements of binding strength, similar to the dissociation constants used by TargetScan-CNN [31].
(2) ‘miRNA target gene prediction’: Given a miRNA and a gene of interest, in a defined biological context specified by the experimental system and cell or tissue type, predict the change in gene expression resulting from perturbation of the miRNA (overexpression or knock-out). The task does not prescribe the information used to generate this prediction; models may incorporate transcript- and isoform-level information, sequence features, expression measurements, or other relevant contextual features, while the prediction outcome remains at the gene level. This task should be evaluated genome-wide and, importantly, for miRNAs with varying levels of conservation, or even artificial miRNA-like sequences. Benchmark datasets should use standardised processing and annotations, retain sufficient metadata to define the biological and perturbation context, and apply leakage-resistant splitting strategies including independent perturbation experiments involving distinct miRNAs. Evaluation should be multifaceted, comparing predictors against experimentally derived ground truth using complementary measures of discrimination, ranking quality, effect-size association, prediction coverage, and recovery of true targets. Comparisons should be performed both on the common set of genes scored by all methods and on each predictor’s full scored set, while also quantifying predictor agreement, and the added value of combining predictors. Formalising this challenge within a robust, transparent and reproducible benchmarking framework will be an important next step.

Treating ‘miRNA binding site prediction’ and ‘miRNA target gene prediction’ as distinct, standardised computational tasks would facilitate a more meaningful comparison of methodological advances and help reduce the ambiguity that currently characterises the field. This would substantially improve our ability to analyse miRNA-mediated regulation at the systems level. Such advances could improve the reliability of miRNA target identification and enable more accurate modelling of miRNA-mediated regulatory networks, thereby providing a stronger foundation for exploring the therapeutic potential of miRNAs. The history of the field suggests that progress has often been driven by improvements in experimental data rather than by algorithmic advances alone. Establishing robust, community-wide benchmarks for these two distinct prediction tasks may therefore be as important for future progress as the development of new deep learning architectures.

## Data Availability

No new data were created or analyzed in this study. Data sharing is not applicable to this article.

## Abbreviations

The following abbreviations are used in this manuscript:

miRNA	microRNA
AGO	Argonaute
UTR	Untranslated Region
CDS	Coding Sequence
HITS-CLIP	High-Throughput Sequencing of RNA isolated by Crosslinking Immunoprecipitation
PAR-CLIP	Photoactivatable Ribonucleoside-Enhanced Crosslinking and Immunoprecipitation
CLASH	Crosslinking, Ligation, and Sequencing of Hybrids
CLEAR-CLIP	Covalent Ligation of Endogenous Argonaute-bound RNAs–Crosslinking Immunoprecipitation
eCLIP	Enhanced Crosslinking and Immunoprecipitation
CNN	Convolutional Neural Network
